# The Relationship Between Internet Addiction and Sleep Disturbance Among Health Sciences Students From Two Universities in Riyadh, Saudi Arabia: A Cross-Sectional Study

**DOI:** 10.7759/cureus.92524

**Published:** 2025-09-17

**Authors:** Meshal M Zuraie, Meshal R Alotaibi, Alaa M Alzamil, Leen F AlSadun, Daniah O Alsayegh

**Affiliations:** 1 Psychiatry, King Abdulaziz Medical City, Riyadh, SAU; 2 Psychiatry, Ministry of Health Holdings, Riyadh, SAU; 3 College of Medicine, King Saud bin Abdulaziz University for Health Sciences, Riyadh, SAU; 4 Psychosomatic and Psychiatry, King Abdulaziz Medical City, Riyadh, SAU

**Keywords:** excessive smartphone use, medical school students, non-substance dependence, pittsburgh sleep quality index (psqi), young’s internet addiction test

## Abstract

Background and aim: Internet addiction (IA) is a compulsive-impulsive spectrum disorder with detrimental effects on social interactions, academic performance, energy levels, and sleep quality. This study aimed to examine the relationship between IA and sleep disturbances among health sciences students at King Saud University and King Saud bin Abdulaziz University for Health Sciences in Riyadh, Saudi Arabia.

Methods: A cross-sectional study was conducted in Riyadh, Saudi Arabia, from April 2023 to October 2023, using a self-administered questionnaire. The survey included demographic questions, the Young's Internet Addiction Test (YIAT), and the Pittsburgh Sleep Quality Index (PSQI). The target sample size was 400 participants. Data were analyzed using chi-square and Fisher’s exact tests, as well as the Mann-Whitney U test. A p<0.05 was considered statistically significant.

Results: The study found that 74.2% of normal internet users were poor sleepers compared to 88.5% of moderate-to-severe internet users. A chi-square test revealed a highly significant association between IA and sleep quality (p<0.05).

Conclusion: The findings of this study highlight the importance of promoting healthy internet use habits and indicate the need for further research into other potential causes of sleep disturbances.

## Introduction

By January 2021, the internet had 4.6 billion users worldwide, serving as a tool for communication, research, socialization, and entertainment. However, excessive use can produce harmful outcomes and is often referred to as internet addiction (IA) or pathological internet use [1]. Sleep quality is shaped by multiple factors, including environmental conditions, lifestyle behaviors, social interactions, and socioeconomic status. Poor sleep is associated with fatigue, reduced concentration, increased stress, and long-term health consequences [2].

The global prevalence of IA has been estimated at approximately 6%, with rates varying widely from 1.6% to 18% across different populations [3]. Individuals aged 15-24 years are particularly vulnerable [3]. Importantly, medical students demonstrate IA rates about five times higher than those in the general population [3], likely due to the unique stressors of medical training, such as prolonged study hours, academic pressure, and heavy reliance on digital resources [4].

One study reported that 5% of respondents had severe IA and 39.6% had moderate IA, with 81.6% of those classified as IA users experiencing poor sleep quality [5]. Younger age, male gender, poor academic performance, home internet access, and frequent chatting or gaming were identified as significant risk factors, while poor sleep quality was strongly correlated with IA [5].

In Saudi Arabia, local data indicate that nearly half of the surveyed students had moderate IA and 2% had severe IA [6]. Over half (54.6%) reported using the internet more than four hours daily, and a similar proportion (51.4%) used it predominantly in the evenings [6]. Such patterns of intensive evening use have been directly linked to poorer sleep quality and adverse health outcomes [7].

Beyond sleep, heavy internet use has also been associated with stress, anxiety, suicidal ideation, and reduced physical activity [8]. Excessive online engagement can further strain family relationships, impair academic performance, and hinder career development due to social isolation, poor time management, and reduced productivity [9]. Accumulating evidence suggests that the association between IA and sleep problems is bidirectional, as excessive internet use can disrupt sleep quality, while pre-existing sleep disturbances may increase susceptibility to compulsive internet behaviors [10,11].

Given these findings, medical and health sciences students represent a particularly at-risk population. This study, therefore, aimed to investigate the relationship between IA and sleep disturbances among health sciences students in Riyadh, Saudi Arabia, and to assess whether the extent of internet dependency was associated with poor sleep quality. This study contributes to the limited literature on this topic, which highlights a high-risk population and may guide future preventive and educational strategies.

## Materials and methods

Study design and participants

This cross-sectional study was conducted between April 2023 and October 2023 among health sciences students at King Saud University (KSU) and King Saud bin Abdulaziz University for Health Sciences (KSAU-HS) in Riyadh, Saudi Arabia.

Inclusion and exclusion criteria

Students were included in this study if they were enrolled in one of six health-related colleges: medicine, dentistry, pharmacy, applied medical sciences, emergency medical services, or nursing. Also, students from all academic years were eligible for inclusion. Students were excluded from this study if they were enrolled outside the health sciences disciplines.

Recruitment and data collection

Data collectors were selected from each university’s health science colleges and invited only their peers to participate. The survey was distributed only in English during the academic year and summer break through two channels as follows: in person on campus and online. No incentives were provided, and no participants were excluded after recruitment.

Sample size calculation

The sample size was estimated using the Raosoft online calculator (Seattle, WA: Raosoft Inc.) for cross-sectional studies, assuming a 95% confidence level, 5% margin of error, and an expected prevalence of 50% to maximize sample size. Although previous studies in Saudi Arabia and neighboring countries reported IA prevalence between 30% and 50% among university students, a conservative estimate of 50% was chosen to ensure the largest required sample size [12]. The minimum required sample was 370, which was rounded up to 400. To account for an anticipated response rate of 20%, at least 1,850 invitations would have been required to achieve the target. Ultimately, 575 students participated through convenience sampling, exceeding the minimum required sample size and thereby improving the precision of the estimates.

Data collection and procedures

The questionnaire had three sections as follows: (1) demographic and academic information - age, gender, university, specialty, and year of study. (2) Young's Internet Addiction Test (YIAT) - a validated 20-item tool rated on a six-point Likert scale with a minimum score of 20. Scores of 20-39 were considered an average user, labeled as mild, 40-69 signifies frequent problems due to internet usage, labeled as moderate, and ≥70 means internet usage is causing significant problems, labeled as severe [3]. However, variations are reported in the literature, such as ≤30 indicating normal use, 31-49 mild, 50-79 moderate, and ≥80 severe [13]. In this study, the former scale was used to stratify internet use, while the latter classification was applied in categorical and association analyses. (3) Pittsburgh Sleep Quality Index (PSQI) - this tool assesses sleep latency, duration, quality, efficiency, disturbances, daytime function, and use of aids. Scores <5 indicate good sleep, while scores ≥5 reflect poor sleep [14].

Validity and reliability

Both instruments showed good reliability in the present study. Cronbach’s alpha was 0.904 for the YIAT and 0.815 for the PSQI, indicating good internal consistency.

Statistical analysis

Data were analyzed using SPSS version 25.0 (Armonk, NY: IBM Corp.). Categorical variables were expressed as frequencies and percentages, and continuous variables as mean±standard deviation or median (interquartile range). Group comparisons were performed using the chi-square test or Fisher’s exact test as appropriate. For 2×2 tables, Yates’ continuity correction was applied. The Mann-Whitney U test was used for continuous variables. Effect sizes were reported using Cramer’s V for chi-square analyses. Statistical significance was set at p<0.05.

Ethical considerations

Ethical approval was obtained from the National Committee of Bioethics of King Abdullah International Medical Research Center (KAIMRC) (approval number: IRB/0446/23). Students’ confidentiality was ensured, and the collected students' data were used by the research team only. All respondents read and signed the informed consent before filling it out.

## Results

A total of 575 students completed the questionnaire. Table 1 presents their demographic characteristics as follows: 62.5% were female and 37.5% male. Nearly half of the sample population (48%) were aged 20-21 years, and about half were in their second or third academic years. With regard to the main purpose of internet use, 49.1% reported entertainment as their primary activity, while 37.8% reported study.

**Table 1:** The demographic responses of the study participants. KSAU-HS: King Saud bin Abdulaziz University for Health Sciences; KSU: King Saud University

Information	Response	Frequency	Percentage	Valid percentage	Cumulative percentage
Gender	Male	215	19.3%	37.5%	37.5%
	Female	359	32.3%	62.5%	100%
Age	18 years	18	1.6%	3.1%	3.1%
	19 years	86	7.7%	15%	18.1%
	20 years	131	11.8%	22.8%	40.9%
	21 years	145	13%	25.2%	66.1%
	22 years	102	9.2%	17.7%	83.8%
	23 years	54	4.9%	9.4%	93.2%
	24 years	22	2%	3.8%	97%
	More than 24 years	17	1.5%	3%	100%
University name	KSU	353	31.7%	61.9%	61.9%
	KSAU-HS	217	19.5%	38.1%	100%
Specialty	College of Medicine	263	23.6%	45.7%	45.7%
	College of Dentistry	33	3%	5.7%	51.5%
	College of Pharmacy	42	3.8%	7.3%	58.8%
	College of Applied Medical Sciences	180	16.2%	31.3%	90.1%
	College of Nursing	45	4%	7.8%	97.9%
	Other College	12	1.1%	2.1%	100%
Which academic year are you in?	First year	44	4%	7.7%	7.7%
	Second year	171	15.4%	29.7%	37.4%
	Third year	122	11%	21.2%	58.6%
	Fourth year	87	7.8%	15.1%	73.7%
	Fifth year	96	8.6%	16.7%	90.4%
	Sixth year	41	3.7%	7.1%	97.6%
	Seventh year	14	1.3%	2.4%	100%
Location of residence	Central Region of Riyadh	210	18.9%	36.5%	36.5%
	Northern Region of Riyadh	120	10.8%	20.9%	57.4%
	Southern Region of Riyadh	43	3.9%	7.5%	64.9%
	Eastern Region of Riyadh	138	12.4%	24%	88.9%
	Western Region of Riyadh	64	5.8%	11.1%	100%
What is the distance between your university and your home?	Less than 20 km	273	24.5%	47.6%	47.6%
	Between 20 km and 40 km	226	20.3%	39.4%	86.9%
	between 40 km and 60 km	65	5.8%	11.3%	98.3%
	More than 60 km	10	0.9%	1.7%	100%
Time taken to arrive at your university in the morning	Less than 30 min	272	24.4%	47.3%	47.3%
	between 30 min and 60 min	220	19.8%	38.3%	85.6%
	between 60 min and 90 min	72	6.5%	12.5%	98.1%
	More than 90 min	11	1%	1.9%	100%
Main purpose of internet use	Study	217	19.5%	37.8%	37.8%
	Work	37	3.3%	6.4%	44.3%
	Entertainment	282	25.3%	49.1%	93.4%
	Other uses	38	3.4%	6.6%	100%
Hours spent using the internet per day	Less than 4 h	47	4.2%	8.2%	8.2%
	Between 4 h and 8 h	308	27.7%	53.6%	61.7%
	between 8 h and 12 h	196	17.6%	34.1%	95.8%
	More than 12 hours	24	2.2%	4.2%	100%

According to Young's Internet Addiction Test (YIAT) categories, the majority of participants (58.3%) fell into the moderate category (Table 2). When scores were dichotomized, 40.7% of students were categorized as moderate-to-severe users (≥50), while 59.3% were classified as normal-to-mild users (≤49) (Table 3).

**Table 2:** Results of YIAT categories of participants. YIAT: Young's Internet Addiction Test

YIAT categories	Number of students	Percentage
Mild (20-39)	203	35.3%
Moderate (40-69)	335	58.3%
Severe (70-100)	37	6.4%
Total	575	100%

**Table 3:** IA categories of participants, based on two subgroups. IA: internet addiction

IA categories	Number of students	Percentage
Normal and mild users (0-49)	341	59.3%
Moderate-to-severe users ≥50	234	40.7%
Total	575	100%

Sleep quality assessment indicated that 80% of participants reported poor sleep, while only 20% reported good sleep (Table 4). A significant gender difference was observed, with females scoring higher on internet addiction than males (Mann-Whitney U test, p=0.008) (Table 5).

**Table 4:** Sleep quality categories of participants. PSQI: Pittsburgh Sleep Quality Index

PSQI score categories	Number of students	Percentage
≤5 (good sleep)	115	20%
>5 (bad sleep)	460	80%
Total	575	100%

**Table 5:** Hypothesis test summary. *The significance level is 0.05; <0.05 indicates a significant value. KSU: King Saud University; KSAU-HS: King Saud bin Abdulaziz University for Health Sciences; YIAT: Young's Internet Addiction Test; PSQI: Pittsburgh Sleep Quality Index

Variables	Comparison	Test	p-Value	Interpretation
YIAT score	Male vs. female	Mann-Whitney U test	0.008*	Significant difference
PSQI global score	Male vs. female	Mann-Whitney U test	0.602	No difference
YIAT score	KSAU-HS vs. KSU	Mann-Whitney U test	0.986	No difference
PSQI global score	KSAU-HS vs. KSU	Mann-Whitney U test	0.068	No difference

Internet addiction and sleep quality were significantly related (Table 6). Among normal-to-mild users (≤49), 74.2% were classified as poor sleepers compared with 88.5% of moderate-to-severe users, χ² (1, N=575) = 16.776, p<0.001, Cramer’s V=0.17, indicating a small-to-moderate effect size. When analyzed across three YIAT categories, poor sleep was reported by 66.5% of mild users, 89.9% of moderate users, and 64.9% of severe users, with a stronger overall association observed, χ² (2, N=575) = 48.729, p<0.001, Cramer’s V=0.29, reflecting a moderate effect size. Finally, the purpose of internet use differed significantly by sleep quality. Among poor sleepers, 41.1% used the internet primarily for entertainment, while 27.5% reported using it for study (p<0.05) (Table 7).

**Table 6:** IA and sleep quality - cross-tabulation results. *P<0.05 indicates a significant value. IA: internet addiction

IA classification	IA category	Good sleep (≤5)	Poor sleep (>5)	Total	Poor sleep percentage	p-Value
Binary cut-off	Normal-to-mild (≤49)	88	253	341	74.2%	<0.001*
	Moderate-to-severe (≥50)	27	207	234	88.5%	
	Total (N=575)	115	460	575	-	
Three categories	Normal (20-39)	68	135	203	66.5%	<0.001*
	Moderate (40-69)	34	301	335	89.9%	
	Severe (70-100)	13	24	37	64.9%	
	Total (N=575)	115	460	575	-	

**Table 7:** Cross-tabulation of gender, IA, and sleep quality vs. the main purpose of internet use. *P<0.05 indicates a significant value. IA: internet addiction

Other variables			Main purpose of internet use				Total	p-Value
			Study	Work	Entertainment	Other uses		
Gender	Male	Count	49	13	132	20	214	<0.05*
		% within gender	22.9%	6.1%	61.7%	9.3%	100%	
		% within main purpose of internet use	22.7%	35.1%	46.8%	52.6%	37.3%	
		% of total	8.6%	2.3%	23%	3.5%	37.3%	
	Female	Count	167	24	150	18	359	
		% within gender	46.5%	6.7%	41.8%	5%	100%	
		% within main purpose of internet use	77.3%	64.9%	53.2%	47.4%	62.7%	
		% of total	29.1%	4.2%	26.2%	3.1%	62.7%	
	Total	Count	216	37	282	38	573	
		% within gender	37.7%	6.5%	49.2%	6.6%	100%	
IA	Normal-to-mild users	Count	133	17	169	22	341	0.367
		% within normal-to-mild and moderate-to-severe categories of IA	39%	5%	49.6%	6.5%	100%	
		% within main purpose of internet use	61.3%	45.9%	59.9%	57.9%	59.4%	
		% of total	23.2%	3%	29.4%	3.8%	59.4%	
	Moderate-to-severe users	Count	84	20	113	16	233	
		% within normal-to-mid and moderate-to-severe categories of IA	36.1%	8.6%	48.5%	6.9%	100%	
		% within main purpose of internet use	38.7%	54.1%	40.1%	42.1%	40.6%	
		% of total	14.6%	3.5%	19.7%	2.8%	40.6%	
	Total	Count	217	37	282	38	574	
		% within normal-to-mild and moderate-to-severe categories of IA	37.8%	6.4%	49.1%	6.6%	100%	
Sleep quality	≤5 (good sleep)	Count	59	5	46	5	115	<0.05*
		% within grouping into poor and good sleep quality	51.3%	4.3%	40%	4.3%	100%	
		% within main purpose of internet use	27.2%	13.5%	16.3%	13.2%	20%	
		% of total	10.3%	0.9%	8%	0.9%	20%	
	>5 (bad sleep)	Count	158	32	236	33	459	
		% within grouping into poor and good sleep quality	34.4%	7%	51.4%	7.2%	100%	
		% within main purpose of internet use	72.8%	86.5%	83.7%	86.8%	80%	
		% of total	27.5%	5.6%	41.1%	5.7%	80%	
	Total	Count	217	37	282	38	574	
		% within grouping into poor and good sleep quality	37.8%	6.4%	49.1%	6.6%	100%	

## Discussion

Approximately half of the participants were aged 20-21 years, suggesting that many were in the middle of their undergraduate studies. This aligns with prior research indicating that young adults, particularly those in medical fields, are more vulnerable to internet addiction (IA) [3-5,15]. Most participants were from King Saud University (61.9%), reflecting the larger enrollment of health sciences students compared to King Saud bin Abdulaziz University for Health Sciences (38.1%).

Nearly half of the students reported using the internet primarily for entertainment, while over one-third used it mainly for study purposes. This highlights the dual role of the internet among health sciences students. Prior studies have linked IA to leisure-driven activities, such as communication, gaming, video streaming, and sexual content [16-18]. Conversely, non-dependent users often describe the internet as a practical tool for academic purposes, which is consistent with the findings of the current study [18].

Gender differences emerged as an important factor. Females scored significantly higher on IA compared to males, though no differences were observed in sleep quality. Previous studies have often reported that males are more susceptible to IA [5,17,19], typically due to online gaming and pornography use [20]. However, the present study aligns with regional evidence, including a systematic review in Gulf Cooperation Council (GCC) countries, which reported higher IA prevalence among females (48% vs. 24%) [12]. In Saudi Arabia, this may reflect contextual factors, such as social and cultural conservatism, environmental conditions (e.g., extreme heat), and limited outdoor facilities, all of which may encourage prolonged online engagement among females.

A significant association was observed between IA and poor sleep quality, consistent with findings from a recent meta-analysis [11]. Effect sizes (Cramer’s V=0.17 for the binary classification and 0.29 for the three-category analysis) indicate that while the relationship is meaningful, other contributors to poor sleep likely exist. Indeed, 74.2% of students with poor sleep were normal-to-mild internet users, suggesting that academic workload, stress, and lifestyle factors may also play a role. This aligns with evidence from Egyptian medical students showing similar patterns of co-occurrence [5]. These results support the view that IA and sleep disturbance are bidirectionally related as follows: excessive internet use can disrupt sleep routines. At the same time, poor sleep may also increase vulnerability to compulsive online behaviors [11].

The strengths of this study include its relatively large sample size (N=575) and its focus on two major Saudi universities, which provide a broader picture of IA and sleep problems among health sciences students. However, limitations should be acknowledged. The use of self-reported measures may introduce recall bias, and the cross-sectional design precludes causal inference. Additionally, the convenience sampling approach limits the generalizability of the results.

Based on these findings, targeted interventions are warranted for Saudi health sciences students. Educational campaigns should emphasize responsible internet use, digital time management, and stress reduction. Educational programs tailored to health sciences students could focus on balancing academic workload, reducing late-night online studying and social media use, and integrating structured relaxation or physical activity sessions into the medical curriculum. In parallel, strategies to improve sleep hygiene should be promoted, including awareness of regular sleep schedules and the use of relaxation techniques. University counseling centers should provide targeted awareness sessions on healthy internet use and its connection to sleep, particularly for students in high-stress specialties. Future research should examine additional contributors to sleep problems, including academic stress, lifestyle behaviors, and mental health status. It should employ longitudinal designs to clarify the directionality of the IA-sleep relationship.

## Conclusions

This study demonstrated a high prevalence of internet addiction and poor sleep quality among Saudi health sciences students, with females reporting higher IA scores than males. A significant association was observed between IA and sleep disturbances, with moderate-to-severe users more likely to experience poor sleep. Nonetheless, a substantial proportion of normal-to-mild users also reported sleep problems, indicating that additional factors, such as academic workload, stress, and lifestyle, may contribute. These findings highlight the bidirectional relationship between IA and sleep quality and underscore the need for tailored educational and counseling programs within universities. Future research using longitudinal designs is recommended to further explore causality and identify strategies to mitigate these interrelated challenges.
